# Successful treatment of early cutaneous squamous cell carcinoma with hypofractionated radiation therapy in an African lion (*Panthera leo*)

**DOI:** 10.4102/jsava.v92i0.2134

**Published:** 2021-06-24

**Authors:** Louise van der Weyden, Nicolize O’Dell, Alida Avenant, Paolo Pazzi, Katja N. Koeppel

**Affiliations:** 1 Wellcome Genome Institute, Wellcome Sanger Campus, Cambridge, United Kingdom; 2 Department of Paraclinical Sciences, Faculty of Veterinary Science, University of Pretoria, Onderstepoort, South Africa; 3 Centre for Veterinary Wildlife Studies, Faculty of Veterinary Science, University of Pretoria, Onderstepoort, South Africa; 4 Department of Companion Animal Studies, Faculty of Veterinary Science, University of Pretoria, Onderstepoort, South Africa; 5 Production Animal Science, Faculty of Veterinary Science, University of Pretoria, Onderstepoort, South Africa

**Keywords:** lion, skin, cancer, radiation therapy, actinic damage, laminar fibrosis, UV

## Abstract

Cutaneous squamous cell carcinoma (SCC) is a slow growing but locally invasive neoplasm, most commonly caused by prolonged exposure to ultraviolet (UV) radiation. Whilst SCC accounts for 15% of skin tumours in domesticated cats, cutaneous SCC in non-domesticated felids (apart from captive snow leopards) appears to be uncommon, with only three reports in the literature to date. In this report, a captive African lion (*Panthera leo*) presented with two ulcerative lesions on the nasal planum. Histopathology of the lesions revealed epidermal keratinocyte dysplasia and neoplastic basal- and supra-basal epithelial cells with dyskeratosis and evidence of basement membrane breaching and dermal invasion, consistent with a diagnosis of SCC. There was also evidence of laminar fibrosis and inflammation of the subjacent dermis suggesting that the SCC most likely resulted from UV-induced neoplastic transformation of the epidermal squamous epithelium following actinic keratosis. The lion was treated with hypofractionated radiation therapy and remained in remission until his death (euthanised 17 months later because of age-related chronic renal failure). This is the first report of cutaneous SCC in a lion with evidence of actinic damage and resolution after radiation therapy.

## Introduction

Squamous cell carcinoma (SCC) is a malignant neoplasm arising from squamous epithelium. Squamous cell carcinomas account for 15% of skin tumours in domestic cats, with most feline cutaneous SCCs occurring on the head, often involving the pinna, eyelid and nasal planum (Miller et al. 1991). In contrast, SCC in non-domestic felids is rarely reported, except in captive snow leopards, where one survey of 424 animals found SCC accounted for 9% of the mortalities (Joslin et al. 2000). In this species, SCC is usually found on the ventral surface of the tongue, face and forelimbs and is mostly associated with papillomavirus-induced malignant transformation (Terio, McAloose & Mitchell 2018). Histopathological changes that support a viral aetiology consist of koilocytes (keratinocytes with shrunken basophilic nuclei surrounded by a clear halo) and/or keratinocytes with increased greyish-blue granular cytoplasm and occasional eosinophilic intranuclear inclusions. This is referred to as viral cytopathology and is associated with a hyperplastic epithelium (Munday 2014).

Excluding snow leopards, reports of SCC in non-domesticated felids have mostly been in the oral cavity (in *Lynx* species [Altamura et al. 2011; Gunson, Klein & Reid 1978; Sladakovic et al. 2016], a North American Amur leopard (*Panthera pardus orientalis*) (Napier et al. 2018) and a Siberian tiger (*Panthera tigris altaica*) [De Oliveira et al. 2018]), with only three reports of cutaneous SCC. Firstly, a 16-year-old female captive tiger (*Panthera tigris*) who had SCC on the left rear limb underwent surgery to remove the mass; however, recurrence was documented 2 years later (Owston, Ramsay & Rotstein 2008). Secondly, a 3-year-old female wild African lion (*Panthera leo*) had severe swelling and draining sinus tracts on the front left paw and the lesions were unresponsive to 7 months of treatment with various antibiotic regimes such that her deteriorating condition resulted in humane euthanasia. At postmortem, SCC was confirmed, presenting as discharging sinuses lined by neoplastic squamous cells (Mwase et al. 2013). Thirdly, a 15-year-old female captive clouded leopard (*Neofelis nebulosa*) had a swelling and an abscess on the right hind paw and biopsy revealed a well-differentiated SCC. Attempts at surgical excision and cryosurgery proved unsuccessful and subsequent recurrence of the mass led to a mid-femoral amputation (Kesdangsakonwut et al. 2014).

Neither actinic damage as the underlying cause of SCC, nor use of hypofractionated radiation therapy as the sole means of successful therapy have been previously reported in any non-domestic felid. The present report describes a SCC on the nasal planum of an African lion that showed concomitant evidence of actinic damage in the surrounding area and successful treatment with radiation therapy.

## Case presentation

**FIGURE 1:**
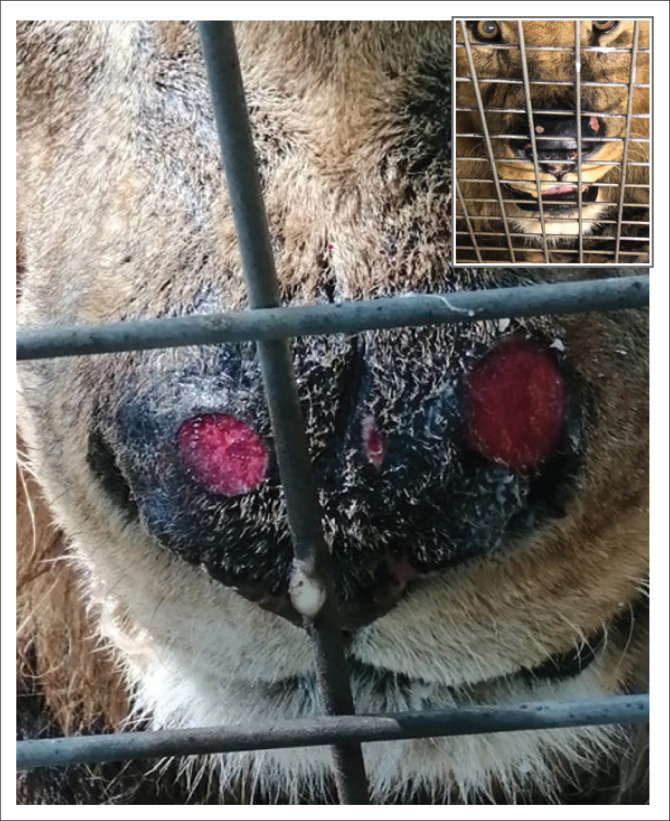
Photograph of the nasal planum of the lion showing two well-demarcated ulcerative lesions. Inset: Nasal planum 2 months after diagnosis and receiving radiotherapy.

A 16-year-old intact male African lion from the Lory Park Zoo and Owl Sanctuary in Midrand, South Africa, who had lived at the property since he was a few days old, was presented to the Wildlife Clinic at Onderstepoort Veterinary Academic Hospital with a 2-month history of two round, well demarcated, laterally positioned, ulcerative lesions approximately 5 cm and 3 cm diameter and a midline elliptical lesion approximately 0.5 cm × 0.2 cm, on the nasal planum (Figure 1). No other abnormalities were reported and all vital parameters were within normal limits. Differential diagnoses considered included trauma, fungal infection, bacterial infection or hypersensitivity to black flies (*Simulium* spp), a common presentation in large captive carnivores in South Africa (Myburgh & Nevill 2003). The lesions were treated daily with oral corticosteroids (Lenisolone, Pharmacare Ltd., Woodmead, South Africa) initially 120 mg once daily for 3 days then tapered (over 8 days) and topical ointment (F10 barrier ointment, Health and Hygiene Ltd., Florida Hills, South Africa) to reduce inflammation and irritation. However, the lesions continued to expand.

**FIGURE 2:**
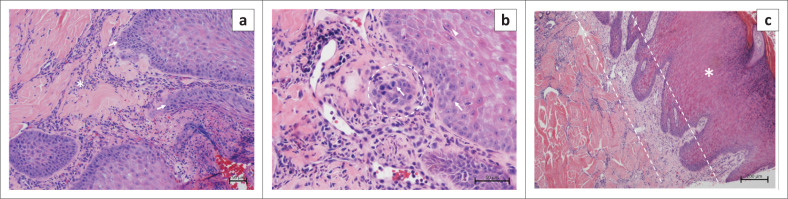
Histological features of the lesions. (a) Epidermal rete peg accentuation (*arrows*) and subepidermal perivascular inflammation (*asterisk*) (HE stain, x100 magnification). (b) Dyskeratosis (*arrowhead*), mitoses (*arrows*) and island of neoplastic epithelial cells below the basement membrane (*dashed circle*) (HE stain, x200 magnification). (c) Acanthosis (*asterisk*) and pale band of subepidermal laminar fibrosis (*area between parallel dashed lines*) (HE stain, x40 magnification).

One month later the 260 kg lion was darted with medetomidine (7 mg, Wildlife Pharmaceuticals, South Africa) and Zoletil (160 mg, Virbac, South Africa) to allow samples to be taken for further analysis. Blood was collected from the femoral vein for routine haematology and clinical biochemistry analysis. The blood sample revealed all parameters to be within normal ranges (Appendix Table 1-A1). Four punch biopsies from the ulcerative lesion on the nasal planum were taken with a 6 mm biopsy punch: one from the centre and one from the periphery of each of the two large lesions. Biopsies were fixed in 10% buffered formalin, embedded in paraffin wax and sectioned for histopathological evaluation. All the sections were stained with haematoxylin and eosin (H&E) and examined by light microscopy by veterinary pathologists (N.O’D. and L.A.). All four of the examined sections revealed similar changes that consisted of moderate acanthosis of the epidermis with mild to moderate parakeratotic and/or orthokeratotic hyperkeratosis. There was evidence of epidermal rete peg accentuation, keratinocyte dysplasia and dyskeratosis and occasional brightly eosinophilic apoptotic cells. The basal- and supra-basal epithelial cells displayed loss of polarity, moderate pleomorphism, hyperchromatic nuclei with 1–2 prominent nucleoli and a mitotic count of 25 in 10 high-powered fields (Figure 2a). In one section, evidence of dermal invasion and breaching of the basement membrane by an island of neoplastic keratinocytes could be observed (Figure 2b). All sections revealed some degree of laminar fibrosis of the subjacent superficial dermis (Figure 2c) associated with mild perivascular inflammation consisting of predominantly plasma cells and lymphocytes accompanied by fewer neutrophils, macrophages and occasional eosinophils. Ulceration of the epithelial surface, with associated pleocellular inflammation and serocellular crusting were present in areas. The diagnosis was SCC with actinic keratosis.

**FIGURE 3:**
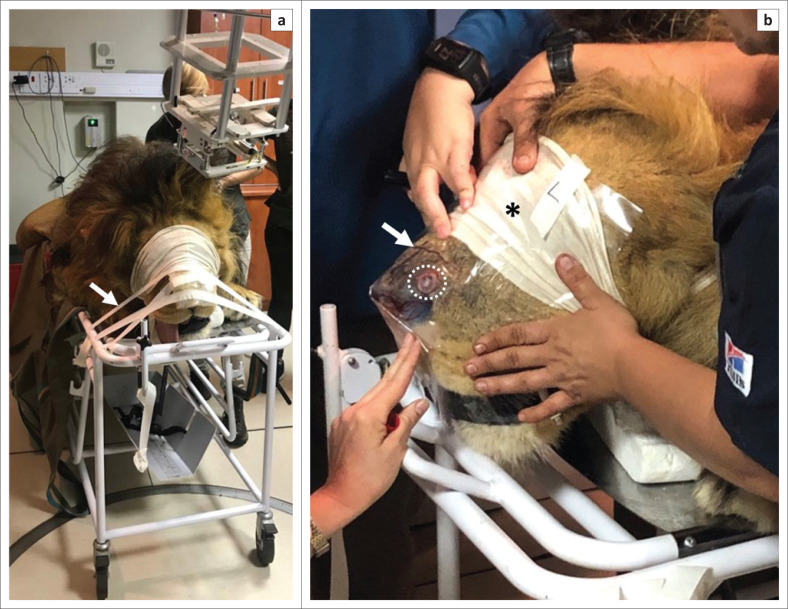
Photograph of the lion being prepared for radiotherapy treatment. (a) Lion strapped onto a patient trolley in the prone position, with the head secured with tape (*arrow*) to prevent movement. (b) Blindfold applied for protection of the eyes (*asterisk*) and Elasto-Gel applied and marked for correct positioning (*arrow*) to ensure the correct dose of radiation was uniformly delivered to the affected area of the nasal planum (*dashed circle*).

The decision was made to use radiotherapy to treat the SCC lesion, as surgical resection was not possible because of its location and cutaneous SCC in domestic cats has been reported to respond well to radiotherapy (Cunha et al. 2010; Gasymova et al. 2017; Theon et al. 1995). In addition, radiotherapy has been previously used in conjunction with immunotherapy and surgical excision in a lion to successfully treat a melanoma of the lip (4 weekly treatments of 8 gray (Gy) external-beam hypofractionated radiation and 4 bimonthly immunotherapy treatments were used to reduce the tumour size by 50%, after which surgical excision was performed) (Steeil et al. 2013). In contrast, a single fraction of 22 Gy using stereotactic radiotherapy was unsuccessful in treatment of a facial leiomyosarcoma in a tiger (*Panthera tigris*), which succumbed to severe metastatic disease 4 months later (Boudreaux et al. 2019). The radiotherapy was performed at the Mediclinic Muelmed Hospital in Pretoria, where the lion was sedated as described here (topped up with intravenously administered 0.5 mg/kg ketamine as needed). The lion was strapped onto a patient trolley in the prone position, with the head secured with tape to prevent movement (Figure 3a) and an Elasto-Gel used to ensure the correct dose of radiation was uniformly delivered to the affected area of the nasal planum (Benson et al. 1991) (Figure 3b). Using an Elekta Synergy machine (Elekta Instrument AB, Stockholm), the lion was administered 8 Gy radiation directly to the lesions (~5 min duration). Upon recovery, the lion was transferred back to Lory Park Zoo and Owl Sanctuary. This procedure was repeated on three further occasions, each 8 days apart. In total, the lion received 32 Gy radiation. During the month of treatment, the lion was kept in the shade in his enclosure (with his female companion). At the end of radiation therapy, a blood sample was taken and all values were again within the normal range (Appendix Table 1-A1).

Follow-up punch biopsies of the nasal planum 14 months later, because of the presence of ulcerating lesions at the site of the original SCC lesion, showed no evidence of neoplastic changes. The new lesions were associated with mild eosinophilic dermatitis, indicative of an allergic reaction of unknown aetiology (possibly an insect bite hypersensitivity reaction). Three months later, his condition deteriorated such that euthanasia was required. Histopathological findings at autopsy suggested chronic renal failure, which was most likely age-related. There were no neoplastic lesions observed in any of the organs examined (kidney, stomach, spleen, heart, liver, pancreas, lymph node, lung, oesophagus and diaphragm).

## Discussion

Older domestic cats are at greater risk of developing SCC, with the average age at presentation being 10–12 years (Hauck 2012; Miller et al. 1991). Factors contributing to the development of cutaneous SCC are prolonged exposure to ultraviolet (UV) radiation, lack of skin pigment and a sparse hair coat (Goldschmidt & Goldschmidt 2017) and infection by papilloma virus. UV radiation can produce deoxyribonucleic acid (DNA) damage either directly or through the production of reactive oxygen species, leading to the activation of oncogenes or inactivation of tumour suppressor genes, which results in the survival and proliferation of the damaged keratinocytes (D’Orazio et al. 2013). Initiation and progression of skin carcinogenesis mediated by UV radiation involve many complex pathways, including proliferation, apoptosis, autophagy, DNA repair, checkpoint signalling, metabolism and inflammation (Kim & He 2014). Feline skin chronically exposed to the sun presents clinically as mild erythema in the affected areas (particularly the ear margins, preauricular areas, periocular areas, nose and lips [Almeida et al. 2008]), which may histologically show preneoplastic and early neoplastic changes. As the lesions worsen, scaling and marginal crusting may form, with extensive ulceration and destruction of the affected areas seen in the later stages (Sherding 1994). Domestic shorthair cats showed a high level of ear-skin hypersensitivity to solar radiation, with a positive correlation also observed between age, degree of oedema and sclerosis in the upper dermis, telangiectasia, squamatisation of basal keratinocytes and epidermal thickness and the degree of photodamage (Almeida et al. 2008). UV exposure can lead to actinic damage known as solar or actinic keratosis, which is a pre-neoplastic lesion composed of dysplastic cells that do not breach the epithelial basement membrane. With a range of presentations from an erythematous, scaly thickening of the skin to shallow, crusting lesions, it typically occurs on lightly haired, non-pigmented skin (Murphy et al. 2013). With prolonged UV radiation, most actinic keratosis lesions progress to invasive SCC (Röwert-Hubert et al. 2007). Here we have described the first report of a non-domesticated felid showing signs of SCC associated with actinic dermatosis, which suggest UV-induced injury resulting in neoplastic transformation. No viral cytopathology was present in this case, therefore making papillomavirus infection, as seen in snow leopards, a less likely aetiology.

Although a range of different treatments have been used in domestic cats with nasal planum SCC such as strontium (Sr90) plesiotherapy (Berlato et al. 2019; Hammond et al. 2007), boron neutron capture therapy (Trivillin et al. 2008) and intralesional carboplatin and superficial radiotherapy (De Vos, Burm & Focker 2004), radiation therapy remains the most common therapy. Several radiation therapy protocols are in use including a Monday–Wednesday–Friday schedule (Theon et al. 1995), hypofractionated (Cunha et al. 2010) and accelerated protocols (Gasymova et al. 2017). In terms of efficacy, the results from different studies reflect the different levels of radiation used; however, in general a tumour response (and manageable toxicity) is observed in most cats, with long tumour control durations achieved in some cats, although complete remission is rare. The cutaneous SCC diagnosed in the lion in this report was successfully treated with hypofractionated radiation therapy and remained disease-free until it was euthanised for age-related chronic renal failure (17 months later).

In conclusion, cutaneous SCC, although rarely reported in non-domesticated felids, should be considered as a differential diagnosis for a long-term, non-healing skin wound in these animals and can be successfully treated with radiation therapy. It is hoped that further investigations of African lions, both in wildlife reserves and zoological facilities, will add to a better knowledge and understanding of cutaneous tumours in this species.

## Appendix 1

**TABLE 1-A1:** Diagnostic laboratory report for the lion, showing haematology, biochemistry and blood gas electrolyte values at the two times blood was taken for analysis.

Test	Units	Reference range	Result (pre-therapy)	Result (post-therapy)
Haemoglobin (Hb)	g/L	85–170	150	139
Red cell count	×10^12^/L	5.3–10.39	9.40	9.53
Haematocrit	L/L	0.250–0.528	0.41	0.43
Mean corpuscular volume	fL	41.2–58.0	43.8	44.9
Mean corpuscular Hb	Pg	14.1–18.4	16.0	14.6
Mean corpuscular Hb concentration	g/dL	28.8–39.0	36.6	32.5
Red cell distribution width	%	15.1–25.6	16.9	16.3
White cell count	×10^9^/L	5.4–18.5	12.27	14.16
Segmented neutrophil	×10^9^/L	0.01–15.92	9.82	11.47
Band neutrophil	×10^9^/L	0.00–1.80	0.49	0.00
Lymphocyte	×10^9^/L	0.00–4.72	0.49	1.98
Monocyte	×10^9^/L	0.074–0.944	0.61	0.28
Eosinophil	×10^9^/L	0.000–1.299	0.86	0.42
Basophil	×10^9^/L	0.000–0.070	0.00	0.00
Platelet count	×10^9^/L	122–555	312	422
Parasite ID	-	-	None seen on smear	None seen on smear
Total serum protein	g/L	53–83	68.6	64.7
Albumin	g/L	N/A	33.9	31.0
Globulin	g/L	21–57	34.7	33.7
A/G ratio	-	0.1–1.4	1.0	0.9
Alanine aminotransferase	U/L	15–109	72.0	45.6
Alkaline phosphatase	U/L	0–57	6	5
Glucose	mmol/L	0.39–12.90	8.3	NT
Serum inorganic phosphate	mmol/L	N/A	1.59	NT
Cholesterol	mmol/L	2.12–6.64	3.3	NT
Urea nitrogen	mmol/L	6.2–31.4	17.6	23.4
Creatinine	umol/L	41–326	246	184
Sodium	mmol/L	136–155	147	149
Potassium	mmol/L	3.1–6.0	4.25	4.74
Chloride	mmol/L	94–130	116.0	NT
Calcium	mmol/L	1.7–3.1	2.42	NT
Ionised calcium	mmol/L	1.13–1.65	1.19	NT
Magnesium	mmol/L	0.37–1.19	1.07	NT

A/G, albumin/globulin; NT, not tested; ID, identification.
